# Identifying and Resolving End of Session Cues in Substance Detection Canine Training

**DOI:** 10.3389/fvets.2018.00206

**Published:** 2018-09-06

**Authors:** Jan Topoleski, Craig A. Schultz, Wynn G. Warren

**Affiliations:** Forensic Canine Program, Laboratory Division/Evidence Response Team Unit, Federal Bureau of Investigation, Quantico, VA, United States

**Keywords:** canine substance detection, canine behavior, substance detection canine training, working canine behavior, end of session cues, poisoned cues, premack principle, counterconditioning

## Abstract

When training and working a substance detection canine, a trained final response should be performed immediately upon recognition of odor (Generally, a 1–3 s window is preferred within our detection practices). Typical canine training places much emphasis on planning and setting up training scenarios to achieve specific objectives but not much consideration is given to how to end a training session. When the canine fails to maintain criteria, trainers are left trying to determine the cause of poor performance. One consideration often overlooked is a phenomenon called End of Session Cueing that may exist in detection training whereby a previously trained canine no longer responds to odor because it has taken on aversive association. This may be due to several factors associated with motivation. The sequence of events at the end of a session can be as equally important to maintain motivation for the task of scent detection in future sessions. This paper will identify and examine multiple factors associated with “End of Session Cues” in working dogs, how they may be responsible for poor final response performance and discuss potential strategies to address them.

## Introduction

The trained final response behavior and how it relates to odor are a culmination of several factors which include but are not limited to individual and canine breed selection, behavioral genetics, trainer and handler skill levels and environmental experience (1, 2). The final response can be any behavior that is trained or conditioned during the initial odor imprinting process commonly associated with detector dog training. The Scientific Working Group on Dog and Orthogonal detector Guidelines (SWGDOG) and the National Institute of Standards and Technology's (NIST) Dogs and Sensors Subcommittee operationally defines imprinting as, “A phenomenon by which an animal, during a formative stage of life, forms a lasting attachment to, and preference for, some object or activity through exposure to the same independent of consequences. Operational usage: A method of initial odor/scent discrimination training” (3, 4).

The type of behavior selected as a final response is usually dependent on the target odor source and the ultimate utilization of the canine. For example, human remains detection canines may be trained to bark upon finding the odor of human remains so as to not disturb potential evidence and to alert handler of the presence of the target odor when out of view. Often times final response behaviors can be breed specific behaviors that naturally occur and are captured, or they are behaviors shaped to meet particular operational requirements.

In substance detection canines, a phenomenon may exist whereby newly trained or experienced canines, have progressed through odor imprinting/association and final response training phases with high rates of success and have demonstrated proficiency in various environments. However, over time as the training is moved to different locations or shifted to different contexts the canine seems to make a conscious decision to approach target odor, investigate the origin of the odor, then ignore it altogether and engage in other activities not associated with odor detection. This occurs even if the canine has had much exposure to a certain type of search environment/context with a high rate of reinforcement.

A seasoned handler or trainer would almost immediately identify the problem as a lack of focus, lack of motivation, or a reduced interest for the task of odor detection. All of these may be correct, however, how often do trainers/handlers observe the sequence of events at the ***end*** of a session and take that into consideration when evaluating these training challenges? This is why it is also important to understand and identify End of Session Cues and how these cues and events can negatively impact performance.

## Discussion

### End of session cues

End of Session Cue(s) (EoSC) is simply a behavior or a series of behaviors or events that has been understood by the animal to mean that the training session is about to end (5). This could be purposely trained, such as a “Free” cue at the end of a training session indicating that the session is complete and the animal is released. Conversely, it can also be a cue that an animal has learned without formal training to associate experiences or certain stimuli with an aversive event. For example, marine mammals can tell by the sound of the trainer's near empty fish buckets that they are almost out of fish (reinforcer). When this happens, behavior performance may decrease as animals may start ignoring trainer cues because they may not have enough reinforcement left. Through association, the animal learns that the trainer is about to leave the area, and ultimately, the animal expects that the session is about to end, which equates to the animal perceiving that opportunities for reinforcement are decreasing or have ended. When this occurs, it may not be worth the animal's effort to continue to perform behaviors or interact with its trainer.

Under these circumstances the animal then finds something else of value in the environment to engage in. This is problematic as the animal then learns to reinforce itself for incorrect or undesirable responses, meanwhile, trained and desired behavior(s) may fade away and ultimately cease to occur if the EoSC is perceived by the animal as an aversive event. Whatever that stimulus in the environment is that the animal engages in may now become a competing stimulus to the task in which the animal was trained to do or asked to do by the trainer. This can then become even more problematic as the undesirable behavior continues to occur with no consequence if the trainer allows the animal to continue to rehearse undesirable behaviors (swimming away, foraging for fish at the bottom of the pool, engaging with other animals in the pool). In the working dog, an example would include not responding to a target odor, only to leave it and engage in a crittering behavior- such as smelling urine spots of other canines or animals, chasing animals, foraging for food, etc. Crittering is defined by SWGDOG and NIST as “A change in the canine's behavior where the canine becomes distracted by animal odor or some other animal distracter. Usually evident as there is a change in body language (head and tail position)” (3, 4).

As these undesirable behaviors continue to occur and are self-reinforced, one must contend with the possibility that the undesirable behavior (chasing animals) may soon replace the desired behavior (locating target odor) during detection tasks. This is due to a component of Hernstein's Matching Law whereby an animal's performance can be directly correlated to the rate of reinforcement the animal received for performing the task in previous trials when presented with choices. When faced with two choices an animal will select the choice that has been reinforced more frequently (6, 7). For the detection canine, if an undesirable behavior that is self-reinforcing (i.e., smelling urine spots) occurs more frequently than responding to target odor, Hernstein's Matching Law would predict that the K9 will most likely choose to engage in the behavior that has the highest rate of reinforcement; even if those behaviors are self-reinforced and undesirable (smelling urine).

In addition, each time undesirable behaviors are allowed to be self-reinforced, the strength of desired behaviors may become reduced and are subsequently either not performed or performed poorly (slower, less efficiently). When desired behavior (responding to target odors) starts to be offered less frequently or not to full criteria, trainers or handlers often fall into the trap of accepting/reinforcing a final response performed at a weaker criteria (slower, less intensity, etc.) simply to end the session. As they continue to reinforce the weakened criteria, it now becomes the new criteria.

This relates to working canines in the following ways; all animals, including canines, learn through cause and effect consequences and associations. If choices are reinforced or punished, behaviors are learned or modified. At a more complex level, we see that not only does learning desired behavior occur through associations, learning of undesirable behaviors can arise through accidental reinforcement. Undesired behaviors can be created in the same way desired behaviors are learned if a trainer fails to realize that they are accidentally reinforcing a behavior (8). For the example, as discussed earlier in which the canine failed to respond to target odor and engaged in crittering behavior instead, the canine self-reinforcing itself for crittering because the trainer failed to acknowledge the undesirable crittering behavior, failed to stop the behavior and still reinforces the canine at the end or the search. Thus, crittering behavior increases.

Just as undesirable behaviors can become established through associations, certain stimuli can also be altered through associations. Stimuli that previously had a positive association can come to have a negative association if paired with something the animal finds aversive. An example for the working dog may include being corrected near target odor, over time, the canine may start to equate target odor with an aversive event and start to avoid target odor all together. Another example may include immediately going back to the vehicle after a successful training session in which the canine found a target odor. In this example, the canine may perceive being returned to the vehicle kennel as a punisher.

A few notes worth discussing are that associations are not always created by singular responses, but can also be made between a series or chain of events. By linking a perceived aversive event (going back to the vehicle) with a previously learned task with positive reinforcement history (odor detection), the canine can develop an aversion that can be associated with a preceding event (interaction during search with handler, responding to directional control commands, etc.), a particular context (odor detection in open area search, wilderness, building, etc.), or even specific reinforcer(s) i.e., ending with a ball as a reinforcer on the last find (thus, deploying the ball may become the EoSC of going back to vehicle).

Additionally, fixed intervals of reinforcement can be inadvertently established through training that does not provide variability in time. This may lead to a decline in performance, however, it is unknown if the concept of “time” can be understood by the canine as an End of Session Cue. For the purposes of this paper, if the odor is perceived as the EoSC, it would be expected that the canine chooses *not* to give its trained final response in order to avoid going back to the kennel, regardless of time.

While not recognized as a technical term, referenced in scientific literature or acknowledged in the field of Behavior Analysis, the concept of “Poisoned Cues” is a term that is used within the animal training industry to illustrate the ability of stimuli that previously had a positive association to take on aversive associations. The term was originally coined by Karen Pryor in 2002 (9, 10). An End of Session Cue can become a form of a poisoned cue if some form of stimuli associated with the ending of a session takes on a negative association. This can also be related to the Premack Principle whereby an aversive activity or association occurs after the performance of a desired behavior thus, weakening it in future trials (11, 12). Use of the Premack principle will be discussed further in the Solutions section of this paper.

Consider the following choices by the canine: either leave odor and extend time in the environment, or, respond to the odor and go back to the vehicle. With that said, if a canine finds immense satisfaction, reward, or reinforcement in hunting or engaging with prey, could it stand to reason that “putting the canine up” at the end of a session for correctly identifying a target odor could be “aversive” or even a mild form of punishment? Granted, we *think we are rewarding* our dog for “the find” by praising, playing, and reinforcing it with what we think is rewarding-which to an extent we are. If one considers the canine that encounters an environment that is rich in stimulation combined with being kenneled all day (lack of activity/stimulation) and a reinforcer that fails to compete with the environment, the choice becomes easy. The canine is likely to value everything else other than the task of odor detection. Are we possibly, inadvertently, negatively punishing the canine by removing a reinforcing stimulus (environment)? Or at a minimum, offering a reinforcer that is of lesser value than the competing environment's value. Is there a way that you can incorporate the environmental experience into your reinforcement? You may also observe that when a session increases in duration or length, performance may drop in anticipation that the session will end and the canine starts to engage in other activities such as crittering. Crittering behavior then competes with target odor because it now becomes more reinforcing than making a find (a find equates to “the session is about to end and they are going back to the vehicle” where no stimulation occurs). Thus, the odor itself becomes “poisoned” and now becomes the EoSC.

In training, often the vehicle is used as a negative punisher in which a canine is placed back in a vehicle for failure to perform a task due to lack of focus or motivation. If the perceived punisher is accidentally applied after a correct response on a target odor, then it would stand to reason that the canine is being punished by going back to the vehicle. Furthermore, Classical Conditioning would show that repeating of this routine would suggest the “target odor” itself may *eventually* trigger a signal that a punisher would be coming soon, thus, giving way for the avoidance of odor and an explanation to why the canine would leave odor to engage in other extraneous behaviors that it finds to be of more value. Consider the following:

$$\begin{array}{l} \text{Negative }\underline{\text{C}}\text{onditioned }\underline{\text{S}}\text{timulusor-CS }(\text{going back to vehicle}\\ =\text{lack of reinforcement or reward}) \end{array}$$

$$\begin{array}{l} \text{Positive }\underline{\text{C}}\text{onditioned }\underline{\text{S}}\text{timulusor}+\text{CS }(\text{odor}\\ =\text{playtime},\text{ interaction},\text{ and stimulation is coming})\\ +\text{CS }(\text{odor})――>\text{-CS}1(\text{vehicle}) \end{array}$$

If reinforcement is weak or not “reinforcing”, ultimately odor may take on the signaling of an aversive event, or, going back to vehicle:

-CS2(odor)――>-CS1(vehicle)

Now that end of session cues or poisoned cues have been discussed and how they can develop, it is important to recognize behaviors associated with them so that we can mitigate them as quickly as possible. Some observable behaviors that handlers/trainers may observe include but are not limited to:

- Approaching, investigating and then leaving a trained odor source to engage in self- reinforcing- play behavior, scavenging or eating in environment, etc.
- “Crittering” behavior (smelling and investigating animal odors or non-targets for an extended amount of time).
- Running off or being unresponsive to the handler while trying to maintain search/directional control.
- Avoidance of the vehicle or hesitation while loading into the vehicle (This may occur during the initial load up or after a successful find).

These are just a few behavioral indicators that may be observed. These may also be observed with other issues as well and should not solely be considered to be end of session cues.

In addition to the behaviors outlined earlier in this article it is equally important to evaluate another part of the equation before determining if end of session cues are a possible culprit of poor training. Consider the canines' daily activity repertoire. If you work with a canine in a professional setting, and perhaps more typical in law enforcement applications, canines may spend a large amount of their day either in a yard, a kennel, a vehicle, or a combination of all. In general, they may only get trained or “worked” realistically for 1–2 h a day. This is actual work time; not putting the dog in the car or truck at the beginning of a shift and driving for 8 h. And if there is inclement weather or an extended lapse in training time, this number will most definitely decrease. This isn't a critique by any means, rather a harsh reality that must be recognized and addressed when training canines. Another important part in this equation is handler action and activity schedules. Consider some of the following questions:

**Handler Action:**

- How is the canine loaded into the vehicleon productive vs. unproductive sessions?
- How is the canine leashed up after each session or search?
- How long do you spend reinforcing the dog before kenneling?
- Is the kennel used as a punisher for poor performance?
- Does your canine get to take its toy with them before the search begins?

**Canine Activity:**

- How long does the canine spend in the kennel between sessions?
- Once the canine is reinforced does your session end immediately or do you continue the search scenario? How often?
- What is the ratio between time spent training or searching, to time spent in the kennel and being inactive in a 24 h period?

An important step in determining the underlying cause of final response regression is to test your assumptions as outlined earlier by setting up controlled field tests and testing the variables that are thought to be at the heart of the issue. The key is to test each assumption or variable separately. The assessment may be best done as part of a team; handler and at least one observer depending on what you are trying to assess. Preferably, the observer will be familiar with the scientific method or conducting blind assessments to help guide the process along and document the observations. To effectively assess the factors noted earlier in this article (behaviors associated with poor motivation, handler action and canine activity) we found it most beneficial to eliminate any extraneous variables that may contribute to the problem, or at least try to account for them when setting up the assessment(s).

### Re-valuate the reward system

It is generally accepted that, some canines, especially those with high prey drive or motivated to chase in the working dog industry, may consider the “act of the hunt” intrinsically reinforcing where no other behavior or object is as reinforcing. Each canine, regardless of breed or litter, may exhibit differences in preference then their closest siblings. This means that even their reward and value systems may be different. To strengthen your reinforcement, consider magnitude or “jackpot” reinforcers and make them an extended “event” not just a short occurrence. This can be accomplished by expanding the reinforcement in both intensity and duration at the end of a session. An example of this would be to reinforce the canine the entire way back to the vehicle, allow the canine to possess the reinforcement on the journey back to the vehicle and continue to praise the canine during the loading process. Emphasis should be placed on the level of arousal that occurs during reinforcement as increased arousal levels have been proven to enhance learning and memory consolidation (13–15). In fact, the reinforcement may not even need to be related to the task of substance detection for learning to occur as the dopamine release during unrelated pleasant experiences affect learning and memory (14, 15).

Something to consider at the end of a training session is occasionally letting the canine engage in extended reinforcement events long enough to reach satiation; letting the canine tell YOU when it is done being reinforced for a change. Engaging in play behavior after a training session has been shown to improve training performance in canines trained in discriminative tasks (16). While the application of play interaction has been historically considered as standard methodology in the substance detection canine training industry due to anecdotal observations; until now, there has been little work completed to quantify the role of playful activity as a reinforcement option in canines trained in discriminative tasks. Another consideration worth mentioning is varying up the type of higher valued reinforcement options. For example, One time the canine gets a lot of praise, receives a ball or tug and the next time it gets reinforced with free time or gets to engage in other behaviors that it values more. The key is getting the canine to associate the higher value reinforcement with the odor and trained final response behavior performance.

The above paragraph simply describes a concept called the Premack Principle whereby one can use an activity or engagement in behavior(s) in which a canine values as a reinforcement option; the more frequent activity will reinforce the other less frequent one (11, 12, 17–19). Premack theorized and proved that if an animal performed a behavior (behavior A) at a greater rate than another behavior (Behavior B), then behavior A can be used effectively as a reinforcer for performing behavior B (12). Lindsay further discusses this by stating the following:

“*During an ordinary training session, the dog is going to prefer performing some exercises more than others. Determining at any moment what the dog would prefer to do and then providing access to that activity on a contingent basis is a sound and efficient incorporation of the Premack principle*.

*It would be consistent with the Premack principle to follow the performance with an even more exciting and reinforcing opportunity.” (11)*.

In working dogs, this was discussed in Schutzhund and protection dogs where the activity that the canine preferred to complete was bite work. The activity of bite work was used to reinforce obedience behaviors (20). If obedience behaviors were performed correctly then the canine was reinforced by being given the opportunity to engage in bite work. In the detection canine, an example of this would include choosing a behavior of higher value that the canine prefers to engage in and using it as a reinforcer for correctly responding to odor. For example, If the canine successfully responds to a target odor it will be given the opportunity to engage in a directional control session. The higher value behavior (directional control) can be independent and semantically unrelated to the lower value behavior (odor detection) so long as it is applied with immediacy, increases emotional arousal and is applied with duration (13, 14, 21, 22).

It is important to note the difference between how Premack principle activities are applied and how specific stimuli are perceived. The Premack principle relates to activities that are performed with frequency and their ability to be used to reinforce or punish behavior, whereas the majority of this paper discusses specific cues associated with performance regression of desirable behaviors. The Premack principle can strengthen or weaken responses based on behaviors performed. Before the activities are applied, specific cues can be associated before the activity is engaged in.

For example, going back to the vehicle (a perceived punisher) is an activity—where the odor becomes the cue that precedes the activity of going back to the vehicle, thus, weakening the trained final response to odor or creating avoidance of the odor altogether.

### Evaluate the canine's routine

As discussed earlier in this paper, the canine's daily routine may not be stimulating enough. By taking a realistic inventory of your dog's daily activity a handler will know how many hours it spends in a kennel, or not training vs. learning and improving? This will be an excellent place to start the problem solving process. In addition, knowing the canine's strengths and weakness can make us aware of the length of sessions that we conduct and compare that to the time that they are alone without stimulation. If we think about the holistic activity of the canine; is the canine getting enough physical or mental stimulation?

### Counterconditioning the odor

While changing the perception of going back to the vehicle from an aversive event to a pleasurable event is necessary so to is counterconditioning the stimulus of the odor to “unpoison” the cue. Taking a few steps back in the training plan to review the odor association or imprinting training may prove to be beneficial. To change the perception of the odor stimulus, repetitiously reinforcing the canine for nose to contact or proximity to the target odor source. By increasing the rate of reinforcement for sniffing the target source without the consequence of immediately returning to the vehicle we are re-establishing the pleasurable experience associated with the detection of target odor. Once the canine demonstrates proficiency of detecting the source of the odor and not leaving it, start incorporating the trained final response criteria. Initially relax the trained final response criteria and increase criteria as the canine demonstrates fluency of the trained final response behavior. It is recommended that while the target odor/cue is being counterconditioned that the canine not immediately be returned to the vehicle upon completion of the exercise so as to not to continue to poison the target odor.

### Evaluate the health and fitness of the canine

Consider moderately exercising the canine prior to a training session or deployment (approximately 1–2 h prior to searching). Exercise benefits include an increase in serotonin and dopamine levels in the brain, which also assist in increased motor coordination, regulation of emotions, and pleasurable feelings (23). In fact, exercise with moderate length and intensity may improve learning and memory consolidation (24, 25). Robbins et al. (26) define fitness for the working dog as a “combination of cardiorespiratory function, balance, strength, flexibility, proprioception, stamina and muscle strength” it is worth noting that exercise should be limited to brief bouts of activity so as not to create fatigue, hyperthermia or impede detection capability. Environmental and physiological factors should be considered and exercise sessions should be structured to slowly increase a canine's fitness level to perform at the level desired. If a canine is not adequately acclimatized to heat and humidity, physical activity in those types of environments can increase heat stress or heat stroke in working canines as they are expected to perform in mentally and physically adverse environments (27).

In addition, be aware of how frequently the canine is working. Keep tabs on the duration of sessions and when your canine “checks out.” It could be that searching has become aversive by reduced motivation over time or fatigue. Perhaps the canine has just been exposed to the same search scenarios and searching isn't as reinforcing, or conversely, that the training is too hard and the canine is perhaps mentally fatigued. An extended break from training and work may help learning in some instances. Rest is a vital component to learning and performance (28).

## Conclusion

The end of session cue is a concept that is often overlooked, underused or even forgotten as a canine's detection proficiency improves. End of session cues can be correlated with the manner in which training sessions end, how and what type of reinforcers are delivered and the manner in which the canines are returned to holding kennels afterwards. Associations of events can be either positive or negative. In the case of poor performance, one should not only evaluate how sessions are conducted but also how they are ended. When evaluating poor performance, improper foundation should be discounted as an underlying cause. Once this is eliminated, the possibility of end of session cues should be explored.

By being aware of the behavior indicators associated with end of session cues and the canine's activity schedule canine handlers/trainers can be more equipped to mitigate their effects on working dog performance.

## Author contributions

JT and CS developed the concept. CS researched the literature support. CS, JT, and WW participated in the manuscript preparation.

### Conflict of interest statement

The authors declare that the research was conducted in the absence of any commercial or financial relationships that could be construed as a potential conflict of interest.
